# Dermatologic Data From the Global Burden of Disease Study 2019 and the PatientsLikeMe Online Support Community: Comparative Analysis

**DOI:** 10.2196/50449

**Published:** 2024-12-11

**Authors:** Mindy D Szeto, Lina Alhanshali, Chandler W Rundle, Madeline Adelman, Michelle Hook Sobotka, Emily Woolhiser, Jieying Wu, Colby L Presley, Jalal Maghfour, John Meisenheimer, Jaclyn B Anderson, Robert P Dellavalle

**Affiliations:** 1 Department of Dermatology, University of Minnesota Medical School, Minneapolis, MN, United States; 2 Department of Dermatology, SUNY Downstate College of Medicine Brooklyn, New York, NY, United States; 3 Department of Dermatology, University Hospitals Cleveland Medical Center, Case Western Reserve University, Cleveland, OH, United States; 4 Department of Dermatology, University of Colorado Anschutz Medical Campus, Aurora, CO, United States; 5 Abrazo Health Network, Phoenix, AZ, United States; 6 College of Osteopathic Medicine, Kansas City University, Kansas City, MO, United States; 7 School of Medicine, Rocky Vista University College of Osteopathic Medicine, Parker, CO, United States; 8 Division of Dermatology, Lehigh Valley Health Network, Allentown, PA, United States; 9 Department of Dermatology, Henry Ford Health, Detroit, MI, United States; 10 Department of Dermatology, Brown University, Providence, RI, United States

**Keywords:** Global Burden of Disease, GBD, PatientsLikeMe, PLM, online support communities, forums, users, social media, internet, demographics, lived experience, disability-adjusted life year, DALY, prevalence, dermatology, comparative analysis

## Abstract

The Global Burden of Disease (GBD) study aims to characterize the worldwide prevalence and morbidity of major diseases, while PatientsLikeMe (PLM) is an online community providing patient-generated insights into lived experiences; for dermatologic conditions, quantitative comparisons of GBD and PLM data revealed expected demographic differences but also notable correlations, highlighting their potential as complementary data sources elucidating unmet patient needs and priorities.

## Introduction

The Global Burden of Disease (GBD) study is a comprehensive epidemiological effort to systematically quantify and study the morbidity and mortality of major diseases by analyzing disease prevalence, risk factors, and outcomes across multiple countries and time periods [1]. Disability-adjusted life years (DALYs) represent total years of life lost to disease and years lived with disability. Many dermatologic diseases are nonfatal but high-burden conditions due to their elevated prevalence and substantial negative quality-of-life impacts; recently, dermatologic conditions became the fourth leading cause of morbidity worldwide [1]. Limited access to specialists, high costs of care, and socioeconomic and geographic disparities compound the burden. Patients’ psychosocial well-being can decline due to the visibility of dermatologic conditions [2].

Thus, examining the lived experiences that patients discuss within online networks such as PatientsLikeMe (PLM) becomes crucial. PLM empowers those with similar conditions to emotionally connect, share information, and build interactive communities. Since 2004, PLM has gained ≥850,000 members reporting ≥2800 health conditions [3], creating a large real-world database of patient-generated information. Given PLM’s popularity, this study and our previous work [4] analyze user demographics and illuminate the daily struggles, treatment challenges, and emotional impact of high-burden dermatologic conditions identified by the GBD. Greater understanding could build awareness of patient concerns, identify trends and unmet needs in disease management, and ultimately contribute to improved patient-centered care and outcomes.

## Methods

Worldwide age-standardized DALYs and prevalence of skin and subcutaneous diseases stratified by sex were obtained from the GBD 2019 [5], as were 95% uncertainty intervals (UIs), capturing uncertainties associated with systematic errors in myriad primary data sources [6]. Total numbers of PLM users, user-reported age, age at first symptom, sex, and diagnosis status was retrieved for each skin disease in April 2023. Nonparametric Spearman correlations (R version 4.2.2; R Core Team) were used to assess the correlation of GBD prevalence and morbidity with PLM user numbers (statistical significance: 2-tailed *P*<.05). To explore differences by sex, *z* tests of proportions were performed for each disease category to compare fractions of men within GBD prevalence values and PLM users who self-reported sex.

All research complied with regulations for the protection of human subjects under 45 CFR 46.104(d) (4), using publicly available data without requiring additional contact or permissions from content creators.

## Results

In the GBD, atopic dermatitis had the highest age-standardized DALYs at 96.7 (95% UI 51.5‐162.6) per 100,000 persons. Fungal skin diseases were most prevalent worldwide (n=578.1 million, 95% UI 521.0‐645.6 million) in both sexes, but men had a slightly higher fraction (51.5%) of total prevalence (Table 1). Acne vulgaris was second-most prevalent (n=231.2 million), followed by scabies (n=187.4 million) and atopic dermatitis (n=171.2 million). Alopecia areata demonstrated the greatest sex difference in GBD prevalence. The PLM psoriasis community had the most users (n=6451), followed by acne vulgaris (n=913) and viral skin diseases (combined users: n=889). No PLM users were found in searches for pruritis and decubitus ulcers. However, Spearman rank-based correlation was statistically significant for the number of PLM disease community users and GBD DALYs (*P*=.04), but not PLM users and GBD prevalence (*P*=.50). Most PLM users self-identified as women, with men comprising only 17.9% (pyoderma) to 46.9% (seborrheic dermatitis). Except for atopic dermatitis, scabies, and seborrheic dermatitis, sex proportions for GBD prevalence and PLM users differed significantly (*z* test: *P*<.05).

**Table 1.** Prevalence and morbidity metrics from the 2019 Global Burden of Disease (GBD) study, numbers of PatientsLikeMe (PLM) users, and percentages by sex, with results of comparative statistical tests. Skin conditions are sorted by highest to lowest disability-adjusted life years (DALYs). The GBD 2019 [5] data query included the following parameters: GBD estimate—”cause of death or injury”; measure—”DALYs”, “prevalence”; metric—”rate, number”; cause—”skin and subcutaneous diseases,” “all subcategories”; location—”global”; age—”all ages”; sex—”both,” “male,” “female”; year—”2019.” Atopic dermatitis, contact dermatitis, and seborrheic dermatitis are components of the “dermatitis” category in the GBD, while pyoderma and cellulitis are subcategories of “bacterial skin diseases.” For the GBD category “urticaria,” autoimmune, cold, cholinergic, solar, aquagenic, and delayed pressure urticaria subtype communities were searched in PLM and the data were combined. Similarly, the GBD category “viral skin disease” comprised PLM chickenpox, herpes zoster, measles, rubella, parvovirus, molluscum contagiosum, and mononucleosis, while the GBD category “fungal skin diseases” included PLM tinea corporis, tinea cruris, tinea capitis, nail fungus, and tinea versicolor. The GBD subcategory for “other skin and subcutaneous diseases” represented over 100 miscellaneous skin conditions listed separately in PLM, and therefore was not queried in this analysis.

Skin condition	GBD age-standardized DALYs per 100,000 persons (95% UI^a^)	GBD prevalence in millions (95% UI)	PLM users, n	Prevalence of men in the GBD (%)	Percentage of men among PLM users (%)	GBD prevalence and PLM user sex proportion: *P* value (*z* test)
Atopic dermatitis	96.7 (51.5‐162.6)	171.2 (164.8‐178.1)	560	40.9	40.4	.80
Acne vulgaris	64.0 (38.5‐101.5)	231.2 (208.2‐255.5)	913	44.4	26.4	<.001
Scabies	62.5 (34.7‐99.9)	187.4 (165.4‐212.1)	80	50.6	39.7	.06
Viral skin diseases	61.1 (39.1‐91.3)	153.8 (148.7‐158.5)	889	51.5	23.1	<.001
Urticaria	50.4 (33.0‐72.2)	65.1 (57.5‐73.5)	444	41.1	31.2	<.001
Psoriasis	45.3 (32.4‐60.0)	40.8 (39.4‐42.1)	6451	50.1	32.9	<.001
Fungal skin diseases	41.7 (17.1‐87.7)	578.1 (521.0‐645.6)	290	51.5	41.6	<.001
Contact dermatitis	29.4 (18.5‐43.9)	91.8 (74.5‐112.6)	16	45.1	18.8	.03
Malignant skin melanoma	22.1 (16.7‐25.8)	2.1 (1.6‐2.6)	440	51.4	36.5	<.001
Pyoderma	21.3 (16.1‐26.0)	46.5 (45.4‐47.6)	85	54.6	17.9	<.001
Pruritus	10.2 (4.9‐18.2)	74.3 (66.4‐83.5)	0	43	—^b^	—
Alopecia areata	7.8 (4.9‐11.5)	18.4 (17.8‐19.0)	282	34.4	28.0	.03
Cellulitis	7.1 (4.9‐8.5)	1.9 (1.8‐2.0)	357	52.4	25.6	<.001
Decubitus ulcer	6.2 (4.8‐7.5)	0.9 (0.8‐0.9)	0	45.6	—	—
Seborrheic dermatitis	4.0 (2.3‐6.3)	22.9 (21.4‐24.3)	368	50.3	46.9	.21

a UI: uncertainty interval.

b Not applicable.

## Discussion

Our GBD-specific findings parallel past results [7]. However [4], numbers and patterns differed between PLM users, GBD disease burden, and prevalence. Varied demographics and data sources could limit comparisons and generalizability; PLM data are self-reported, and PLM reflects online health communities and social media in having predominantly English-speaking female users with internet access [8]. Suggested positive correlations between use and women reporting fair or poor health and comorbidities [9] may partially explain disease-specific *z* test findings. Conversely, the GBD synthesizes census, registry, and other epidemiological data to broadly capture disease prevalence. Our comparative GBD and PLM findings might therefore be biased by disease awareness, diagnostic accuracy, and reporting quality. GBD categories are limited and aggregated (eg, “viral skin diseases”); thus, data from many PLM communities not explicitly delineated by the GBD (eg, lupus, rosacea, cutaneous T cell lymphoma) were combined for comparisons. However, while PLM data do not reflect global burden, our rank-based correlations still suggest potential associations between GBD morbidity and PLM user numbers, highlighting PLM’s potential for complementing epidemiologic data. Future integration of patient-generated data could add nuanced insight into patient experiences and needs, thereby empowering targeted care [10].

## Abbreviations

DALY: disability-adjusted life year
GBD: Global Burden of Disease
PLM: PatientsLikeMe
UI: uncertainty interval

## References

[1] Karimkhani C, Dellavalle RP, Coffeng LE (2017). Global skin disease morbidity and mortality: an update from the Global Burden of Disease Study 2013. JAMA Dermatol.

[2] Ahmed A, Leon A, Butler DC, Reichenberg J (2013). Quality-of-life effects of common dermatological diseases. Semin Cutan Med Surg.

[3] About: empowering patients through community. PatientsLikeMe.

[4] Szeto MD, Hook Sobotka M, Woolhiser E (2024). PatientsLikeMe and online patient support communities in dermatology. JMIR Dermatol.

[5] Global health data exchange GBD results tool. Institute for Health Metrics and Evaluation.

[6] Mathers CD, Salomon JA, Ezzati M, Lopez AD, Mathers CD, Ezzati M (2006). Global Burden of Disease and Risk Factors.

[7] Yakupu A, Aimaier R, Yuan B (2023). The burden of skin and subcutaneous diseases: findings from the Global Burden of Disease Study 2019. Front Public Health.

[8] Sadah SA, Shahbazi M, Wiley MT, Hristidis V (2015). A study of the demographics of web-based health-related social media users. J Med Internet Res.

[9] Nikoloudakis IA, Vandelanotte C, Rebar AL (2018). Examining the correlates of online health information-seeking behavior among men compared with women. Am J Mens Health.

[10] Johansson V, Islind AS, Lindroth T, Angenete E, Gellerstedt M (2021). Online communities as a driver for patient empowerment: systematic review. J Med Internet Res.

